# Internship Preparatory Clinical Course: A Timed-Station Approach to Bridging the Theory-to-Practice Gap

**DOI:** 10.7759/cureus.54662

**Published:** 2024-02-21

**Authors:** Ahmad Alrahmani, Fayez G Aldarsouni, Ghada I Alothman, Norah M Alsubaie

**Affiliations:** 1 Department of Surgery, King Saud University Medical City, King Saud University, Riyadh, SAU; 2 Department of Trauma Surgery, King Saud Medical City, Riyadh, SAU

**Keywords:** pocus (point of care ultrasound, foley carheter, objective structured practical exam (ospe), objective structured clinical examination (osce), transition to internship, surgical simulation, simulation in medical education, internship preparation, medical errors

## Abstract

Background: Medical students' transition to internship has a discernible gap in structured preparation, particularly in practical skill application. We introduced the internship preparatory clinical course (IPCC) to address this gap.

Methods: The course was conducted at the clinical skills and simulation center at King Saud University Medical City and included a total of eight skills distributed across four stations. It employs a timed-station methodology, inspired by the Observed Structured Clinical Examination, but innovatively adapted as a teaching method. Participants were exposed to various stations, such as suturing techniques, interactive mannequins for anatomical structure demonstration, real ultrasound machines on simulated patients, IV cannulation, and urinary catheterization. To facilitate active learning, participants received course materials a day prior, equipped with QR codes for quick reference. Instructors emphasized on-the-spot review, fostering an environment where learners actively engage. Toward the end of the course, after internship a follow-up survey was administered to obtain feedback, achieving a response rate of 83% (n=45/54).

Results: Feedback from the course was overwhelmingly positive, with 91.1% (n=41/45) rating the course as 7 and above out of 10. Participants expressed a higher degree of confidence in providing wound care (Median: 8, IQR: 2) and inserting or removing a Foley catheter (Median: 8, IQR: 4). Lower confidence was observed in stoma examination and care (Median: 5, IQR: 4). During their internships, participants reported that 100% (n=45/45) utilized suturing skills, 48.9% (n=22/45) performed focused assessment with sonography in trauma (FAST) examinations, and 62.2% (n=28/45) attempted nasogastric tube insertions. Additionally, 88.9% (n=40/45) performed wound examinations, 77.8% (n=35/45) provided wound care and dressing, 37.8% (n=17/45) performed abscess drainage, 51.1% (n=23/45) removed and 37.8% (n=17/45) inserted a Foley catheter, and 20% (n=9/45) provided stoma care.

Conclusion: The IPCC effectively addresses the existing gap in medical education, bridging the theory-to-practice divide. The innovative use of the timed-station approach emphasizes the importance of active learning. Our results signify the importance of simulation training, as most interns acknowledge the positive impact of the course on their internship. We recommend preparing pre-interns for internships by giving special consideration to the procedural aspects as most associated with medical errors. The timed-station approach can improve cost-effectiveness and enhance responsibility-driven learning.

## Introduction

The internship year is an important part of undergraduate education. It is a transitional zone to integrate newly qualified physicians into team-based care [1]. Concerns are arising about the competency of these medical graduates [2-5]. This comes in the light of using responsibility-driven learning as a part of intern training [1]. A balance between not compromising patient care and training new physicians is necessary [4,6]. Teams often experience difficulty in integrating new interns [1]. New interns often lack confidence in their ability to perform skills [5]. This manifests at the beginning of the internship. The beginning of July co-occurs with the trainee changeover in most countries. It is commonly believed that health care gets compromised at that time [4,6]. This phenomenon is commonly described as the 'July Effect'. Although we didn't investigate this phenomenon in our center as reported in other centers [6], we, as clinicians, noted a surge in medical errors in July in our hospital. King Saud University Medical City (KSUMC) is a major academic center that provides training for interns coming from a diverse number of medical schools inside and outside Saudi Arabia (SA). The internship year in KSUMC consists of 12 months distributed as follows: two months of ob-gyne and its subspecialties, two months of pediatrics and its subspecialties, two elective months, two months of surgery and its subspecialties, two months of internal medicine and its subspecialties, and two months of emergency medicine and ICU. In SA, prior to beginning their internship year, newly graduated medical students do not receive any specialized training apart from what they learned during their medical studies. After finishing medical school, interns in SA have one week of vacation time. We took this as an opportunity to train interns in basic skills that interns will most commonly encounter. We intentionally targeted skills that fall within the interns' responsibilities and are most commonly associated with medical errors [1,3]. In the literature, several reports discussed courses and boot camps that targeted preparing medical students for internship, we found them to be either theoretically focused or difficult and costly to be implemented for a large number of participants [4,5,7,8]. To our knowledge, there are no reports on courses that target interns' procedural skills abstractly. We want to report our experience developing an Internship preparatory clinical course (IPCC), aiming to create a clear, concise structure of an intern-targeted clinical skills course. Integrating principles of the "Shows How" stage of Miller's pyramid into our methodology [9].

## Materials and methods

The course was conducted on June 22, 2022, one week before the start of the internship, at the Clinical Skills and Simulated Center (CSSC) at KSUMC. The course was conducted in a single day with a total duration of eight hours, from 7:30 AM to 3:30 PM, including a 30-minute registration period and a 30-minute lunch break (Table 1).

**Table 1:** Course Timeline Provided to Participants FAST: Focused assessment with sonography in trauma; NGT: nasogastric tube; IV: intravenous

07:30 AM- 08:00 AM	Registration		
	Group A	Group B	Group C
08:00 AM - 11:30 AM	Basic Suturing, Wound Care and Dressing, Stoma Care and Examination		
	Group D	Group E	Group F
08:00 AM - 09:10 AM	FAST	Airway Management/NGT	IV cannulation/Urinary catheterization
09:10 AM - 10:20 AM	Airway Management/NGT	IV cannulation/Urinary catheterization	FAST
10:20 AM - 11:30 AM	IV cannulation/Urinary catheterization	FAST	Airway Management/NGT
11:30 AM -12:00 PM	Break time		
	Group D	Group E	Group F
12:00 PM - 03:30 PM	Basic Suturing, Wound Care and Dressing, Stoma Care and Examination		
	Group A	Group B	Group C
12:00 PM - 01:10 PM	FAST	Airway Management/NGT	IV cannulation/Urinary catheterization
01:10 PM - 02:20 PM	Airway Management/NGT	IV cannulation/Urinary catheterization	FAST
02:20 PM - 03:30 PM	IV cannulation/Urinary catheterization	FAST	Airway Management/NGT

**Figure 1:**
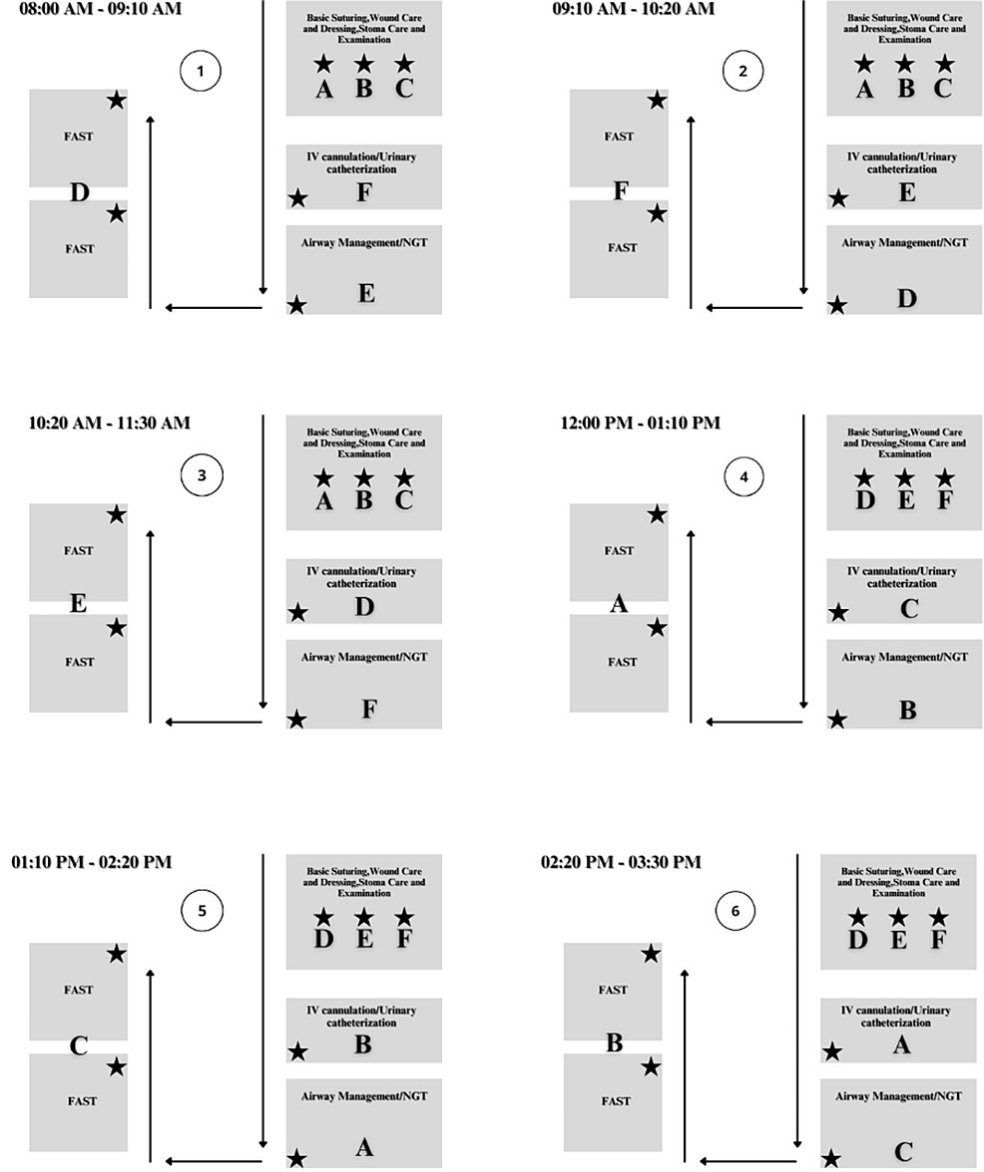
Map of Course Implementation Settings A timed-station approach was necessary to maintain a low participant-instructor ratio. (Black Star: Instructor). Groups were arranged alphabetically.

The course was designed to accommodate 60 participants in total, distributed into six groups of 10 participants each. However, three participants were absent. On the day of the course, participants were given a participating card that contained a QR code for the handouts for all stations. Participants were instructed on how to access these handouts when needed. A timed-station approach was used, with groups rotating between skills when a bell rang. The course included a total of eight skills distributed across four stations. Station 1 included basic suturing, wound care and dressing, stoma care, and examination. Station 2 focused on assessment with sonography in trauma (FAST). Station 3 covered intravenous (IV) cannulation and urinary catheterization, while Station 4 covered basic airway management and nasogastric tube (NGT) insertion/retrieval. Except for Station 1, which had a duration of 3 hours and 30 minutes, all remaining stations were conducted in 1 hour and 10 minutes. The duration of each station was intentionally tailored to the appropriate time needed for the included skills taking the timed-station approach into consideration (Figure 1).

**Figure 2:**
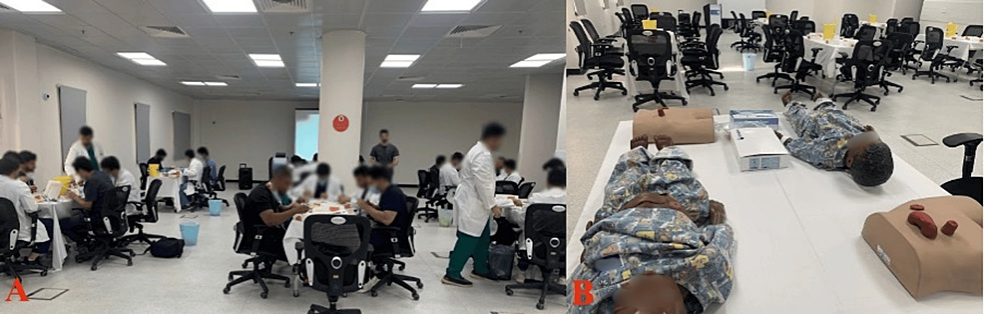
Training Hall for Basic Suturing, Wound Care, Dressing, Stoma Care, and Examination Skills Practice (A) Instructors rotating between two tables, proceeded by a slideshow presentation demonstrating suturing techniques. (B) Stoma mannequins displaying various stoma configurations (End, Loop).

Station 1 was conducted in a hall with eight tables, with five suture kits (Figure 2A) and five wound care kits on each of six tables. The remaining two tables had 10 stoma bags and four stoma mannequins (Figure 2B). The instructor-participant ratio in this station was 1:10, with each instructor rotating between two tables. In the first 15 minutes, a lecture was presented for all participants on simple interrupted, continuous, subcuticular, vertical, and horizontal mattress suturing techniques. This was followed by hands-on practice on the suture techniques under supervision.

**Figure 3:**
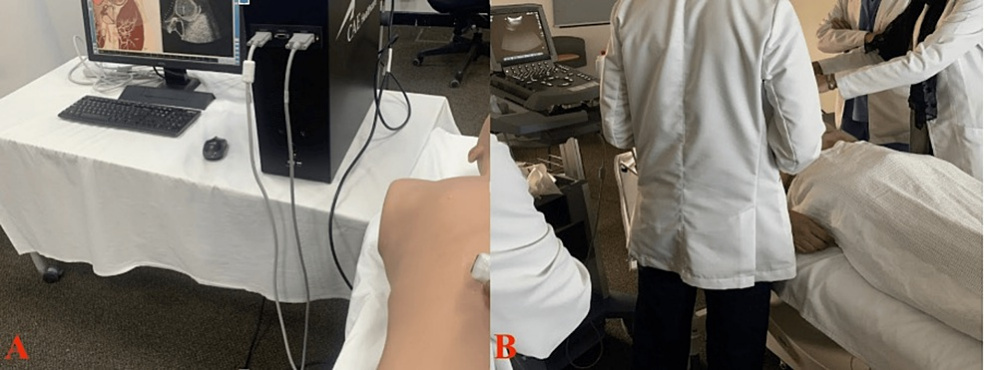
Room Setup for Ultrasound Skill Training and FAST Examination (A) An interactive ultrasound simulator mannequin is used to demonstrate normal anatomical structure. (B) Participants attempt to use a real ultrasound machine on a simulated patient. FAST:  Focused assessment with sonography in trauma

Station 2 was conducted in two separate rooms with one instructor in each, one of which had a simulated ultrasound (US) machine that interacted with participants, presenting anatomy on a screen when the US probe touched a certain location on the mannequin (Figure 3A). The second room had a simulated patient (SP) and a real US device (Figure 3B). After 35 minutes, half of the group rotates, with the remaining half between the two rooms. This station is conducted in an hour and 10 minutes, with an instructor-participant ratio of 1:5.

**Figure 4:**
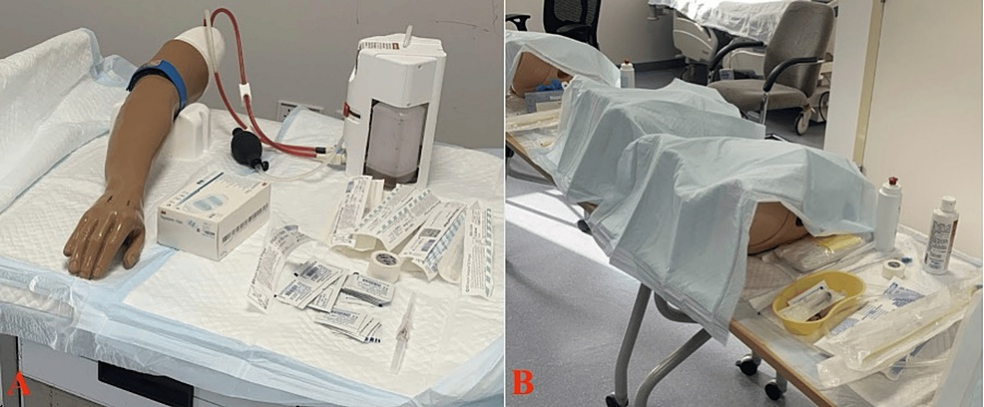
A Picture From the Room in Which IV Cannulation and Urinary Catheterization Were Demonstrated (A) IV cannulation was demonstrated using an arm mannequin. (B) Participants attempted to insert and remove Foley catheters in female and male urinary catheters mannequins. IV: Intravenous

**Figure 5:**
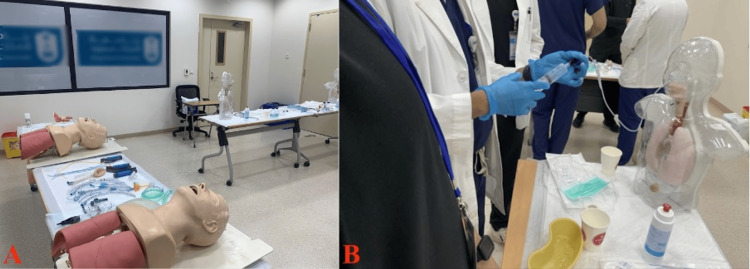
Training Room for Demonstrating Intubation, Airways Management, and NGT Skills (A) Airway management training using mannequins equipped with various types of airways. (B) Participants practicing NGT confirmation with a syringe. NGT: Nasogastric tube

Station 3 (Figure 4A) contained four IV cannulation mannequins and (Figure 4B) four urinary catheterization mannequins. Station 4 contained three mannequins for airway (Figure 5A) management and 3 for NGT (Figure 5B). Basic airway components were covered, including assessment, maneuvers, bag-mask ventilation, and nasopharyngeal, oropharyngeal, and endotracheal intubation skills. Both Station 3 and Station 4 were conducted in 1 hour and 10 minutes in a single room each, with an instructor-participant ratio of 1:10.

**Figure 6:**
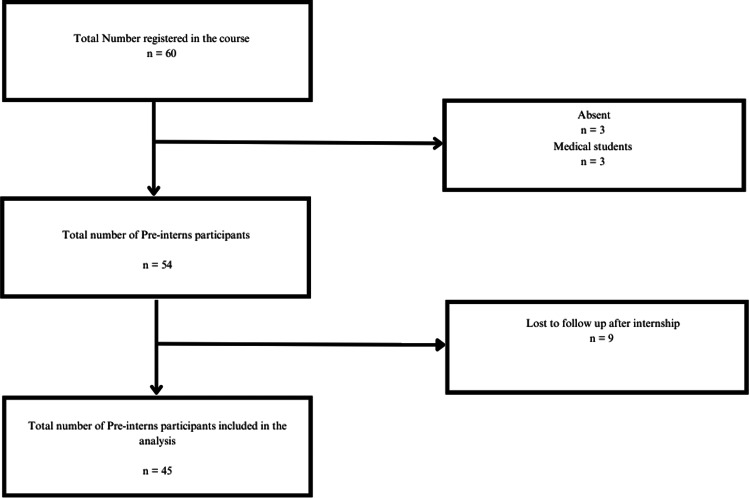
Flowchart of Participant Inclusion for Follow-up

We contacted intern participants of the course toward the end of the internship year to obtain feedback on the course components and whether they had benefited from the skills learned in this course by administering a survey. We achieved a (45/54, 83%) Response rate (Figure 6). 

The statistical analysis for the administered survey was conducted using R statistics software (R Foundation for Statistical Computing, Vienna, Austria). The following parameters were included in the Boxplots Figures that were visualized based on (minimum, Maximum, Interquartile range, Median, 25th Percentile, and 75th percentile) using the ggplot2 package (v3. 3.3; Wickham, 2016). Data in frequency tables were reported as count (Percentage).

 The administrated survey and the study protocol were approved by the Institutional Review Board (IRB) at the College of Medicine, King Saud University (No.E-23-8001).

## Results

Participants' gender distribution and skills utilization during the internship

One hundred percent of participants (n=45/45) had used suturing skills during the internship at some point; 48.9%(n=22/45) had performed a FAST examination, 62.2% (n=28/45) had attempted to insert NGT while 42.2% (n=19/45) had retrieved at least one; 88.9% (n=40/45) of participants performed wound examination, and 77.8% (n=35/45) of participants provided wound care and dressing while 37.8% (n=17/45) performed abscess drainage. Of participants, 51.1% (n=23/45) removed and 37.8% (n=17/45) inserted a Foley catheter while 20% (n=9/45) provided stoma care or examination (n=15/45, 33.3%), 28.9% (n=13/45) of participants retrieved lines, and 33.3% (n=15/45) performed intravenous cannulation, 24.4% (n=11/45) provided basic airway management and 31.1% (n=13/45) had performed or assisted in performing intubation (Table 2).

**Table 2:** Participants' Gender Distribution and Skill Utilization During Internship *"*N" represents the count of participants who reported each variable, and "%" indicates the corresponding percentage of the total participants (n=45) Basic airways include the oropharyngeal airway, nasopharyngeal airway, head-tilt, chin-lift techniques, and Heimlich maneuver. NGT: Nasogastric tube; FAST: focused assessment with sonography for trauma

Variable	(N=45)	%
Gender		
Male	30	66.66%
Female	15	33.33%
Skills Attempted During Internship		
Suturing Skills	45	100%
Performed FAST Examination	22	48.9%
Attempted NGT Insertion	28	62.2%
Retrieved NGT	19	42.2%
Performed Wound Examination	40	88.9%
Provided Wound Care and Dressing	35	77.8%
Performed Abscess Drainage	17	37.8%
Removed Foley Catheter	23	51.1%
Inserted Foley Catheter	17	37.8%
Provided Stoma Care	9	20%
Performed Stoma Examination	15	33.33%
Retrieved Lines	13	28.90%
Performed Intravenous Cannulation	15	33.33%
Provided Basic Airway Management	11	24.4%
Preformed or Assisted in Intubation	13	31.3%

Participants previous surgical experiences and specialty of interest

22.2% (n=10/45) participants had previous surgical course experience attempting to do a Basic Operative Surgical Skills course (BOSS) or Basic Suturing Course (BSC). Most of the participants had the intention to have a career in a surgical specialty, including 17.7%(n=8/45) in general surgery, 8.9% (n=4/45) in orthopedic surgery, 6.67% (n=3/45) in ENT, and 2.2% (n=1/45) in ophthalmology. 8.9% (n=4/45) were interested in emergency medicine, while the remaining participants (n=25/45, 55%) were interested in other nonsurgical specialties (Table 3).

**Table 3:** Participants Previous Suturing Course Experience and Specialties of Interest "N" represents the count of participants who reported each variable, and "%" indicates the corresponding percentage of the total participants (n=45) BOSS: Basic Operative Surgical Skills course; BSC: Basic Suturing Course

Variable	(N=45)	%
Had Previous Suturing Course Experience (BOSS or BSC)	10	22.2%
Specialty of Interest		
General Surgery	8	17.7%
Orthopedic Surgery	4	8.9%
ENT	3	6.67%
Ophthalmology	1	2.2%
Emergency Medicine	4	8.9%
Interested in other nonsurgical specialties	25	55%

Degree of confidence in performing skills and participants' opinion on the course

**Figure 7:**
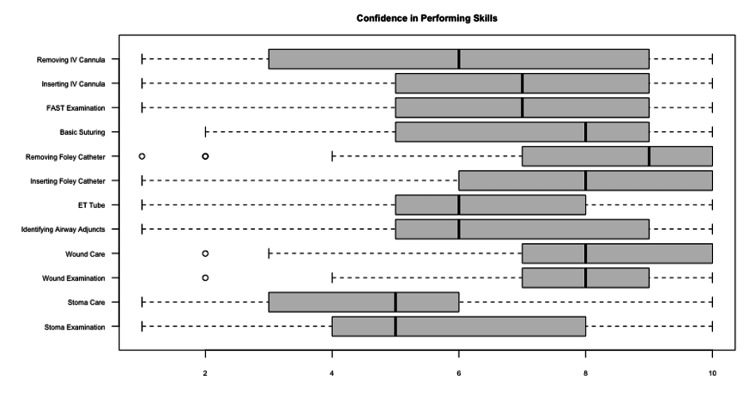
Horizontal Boxplots Reflecting Participant Confidence in Skills Practiced During the Course Each boxplot represents the median confidence level (thick line inside the box), with the bounds of the box depicting the interquartile range (IQR). Whiskers extend to the minimum and maximum values within 1.5 IQR from the quartiles, and outliers are represented as individual points.

Participants have a higher degree of confidence in providing wound care (Median: 8, IQR: 2) and removing (Median: 9, IQR: 3) or inserting a Foley catheter (Median: 8, IQR: 4). Lower degrees of confidence were observed in providing stoma examination and care (Median: 5, IQR: 4) and (Median: 5, IQR: 3) respectively. The remaining skills are presented in Figure 7.

**Figure 8:**
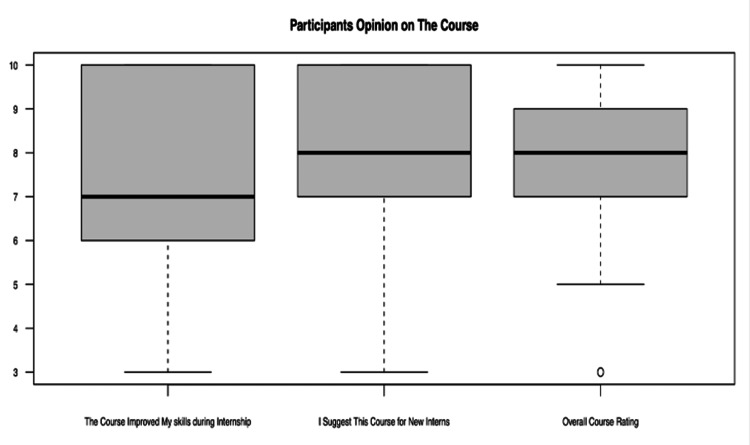
Vertical Boxplots of Participants' Opinions on the Course Each boxplot represents the median confidence level (thick line inside the box), with the bounds of the box depicting the interquartile range (IQR). Whiskers extend to the minimum and maximum values within 1.5 IQR from the quartiles, and outliers are represented as individual points.

The course received good feedback from participants, with (n=41/45, 91.1%) rating the course as 7 and above out of 10. When asked regarding the degree to which they would suggest this course for Pre-interns, (n=37/45, 82.2%) chose 7 and above. (n=34/45, 75.5%) gave the course 7 and above when asked about the degree to which this course helped improve their skills for the internship. Figure 8 presents the components of the course feedback and responses.

## Discussion

Miller's pyramid of competence and its relevance to medical education

Miller (1990) delineated the stages of clinical competence through a structured model termed Miller's Pyramid of Competence [9]. This model comprises four distinct levels: 'Knows' A foundational stage emphasizing the acquisition of basic medical principles, theories, and facts. 'Knows How' This stage involves the application of foundational knowledge to theoretical scenarios. 'Shows How' is characterized by demonstrating skills within a controlled setting. 'Does' Representing the pinnacle of the pyramid, this stage focuses on executing skills with an emphasis on judgment and adaptability. While Miller's framework was primarily contextualized within the assessment contexture, the present study discerned its relevance throughout the journey of a medical student, culminating in their transition to an internship role. The primary objective of this research was to articulate a clear framework for an internship preparatory course. Following the course's deployment, a longitudinal one-year assessment was conducted to evaluate the transference efficacy of the imparted skills during the internship phase. Based on Miller's (1990) framework, the initial years of medical school appear to primarily focus on the 'Knows' stage, emphasizing foundational knowledge acquisition [2]. In contrast, the subsequent clinical years seem to delve deeper into the "Knows How" stage, emphasizing the application of this knowledge. However, a notable deficiency was observed in the structured preparation of medical students transitioning to internships [4]. This deficiency becomes particularly evident in the 'Shows How' stage, where practical demonstrations in controlled settings are crucial [9]. This gap in transition is further accentuated by the well-documented 'July effect', highlighting the challenges new interns face [6].

Utilization of the timed-station methodology: a literature review of other reported courses

A comprehensive literature review yielded limited paradigms focusing on an interactive course structure. Two studies, in particular, described an internship preparatory course where procedural aspects were embedded, covering other aspects such as communication skills [10,11]. However, the time settings, capacity, and outcome were not completely described. Other documented courses in the literature predominantly emphasized the initial two stages of Miller's pyramid [4,5]. Consequently, we conceptualized a novel approach taking into account various factors, including financial implications and frequently encountered skills. The timed-station methodology emerged as a potent tool for implementation. The concept of timed stations traces its roots back to multi-station laboratory tests Objective Structured Practical Examination (OSPE), which later gave rise to the Observed Structured Clinical Examination (OSCE), a widely accepted method for assessing clinical skills [9]. What sets this study apart is its innovative use of this concept: instead of solely using it as an assessment tool, it is employed as a teaching method. The reasons for adopting this approach are multifaceted. As instructors, we believe in active learning by mimicking the real-world challenges interns face, especially when time is of the essence as proposed by Pereira et al. [7]. To facilitate this, participants were provided with course materials a day prior, equipped with QR codes for quick reference on the participants' cards. Instructors emphasized on-the-spot review, cultivating an environment where learners take charge of their education. We purposefully introduced this teaching method to boost interactions and engagement during the stations in case a participant was unprepared for the course.

Integration of wound and stoma care skills

Wound and stoma care and examination skills were intentionally incorporated into the curriculum in recognition of the fact that a dedicated Wound, Ostomy, and Continence nurse and Ostomy Management Specialist - healthcare providers who are certified in the management and care of ostomies - may only sometimes be available in all hospital settings. The absence of specialized stoma care providers can pose significant challenges, not only in the delivery of clinical care but also in terms of patient teaching for self-management [12]. Given that interns frequently rotate through various hospitals - including these without a dedicated stoma nurse or with limited resources for patient education - equipping interns with these essential skills becomes critically important. This training ensures that all patients who undergo ostomy procedures receive proper care and instruction, regardless of staffing variations across hospitals.

Incorporation of FAST and ultrasound basics

We tailored our curriculum to introduce participants to the FAST and the fundamentals of ultrasound handling and anatomy. Acknowledging that a comprehensive mastery of ultrasound techniques cannot be achieved within a brief 70-minute session, we nevertheless deemed it essential to provide an introductory overview. To acquaint interns with its basic application, particularly in urgent trauma assessments. A relative simplicity with which interns can identify free fluid in the abdomen and chest - an often critical and emergent task in trauma care was the main reason this component was added. To ensure the effectiveness of this educational segment, trauma fellows, with their specialized expertise, were engaged to deliver this station. Point-of-Care Ultrasonography (POCUS) is a more comprehensive course covering all aspects of ultrasound handling [13]. Considering the limited time frame, proper probe selection, positioning, image optimization, basic anatomy, and how to identify a positive finding were the only components introduced to participants.

Incorporation of suturing skills into the curriculum

Incorporating suturing skills into the course was a strategic decision, with substantial time allocated to this essential competency, given that surgical rotations are an essential component of the internship year. Previous research has demonstrated that in-situ surgical teaching can significantly prolong operative time, contributing to an estimated annual expenditure of $60 million, as observed in a study examining residents' training [14]. Such costs can deter surgeons from offering basic surgical teaching. Furthermore, interns who lack fundamental surgical skills before entering surgical rotations encounter significant obstacles, which can impede their learning and proficiency. This deficiency affects not only immediate surgical outcomes but also long-term implications, potentially influencing interns' specialty choices based on their experiences during the internship. The reported literature agrees that residents can effectively teach basic surgical skills in simulation centers [15]. In this course, all instructors were general surgery specialists. If this course were implemented in a setting where residents are instructors, we recommend decreasing the participants-instructor ratio by adding more residents to 5:1. Specifically, training residents prior to incorporate time to task.

Limitations and future research directions

In implementing this course, we were mindful of the associated costs. Given that our course primarily targeted recent graduates, we initially hesitated to proceed due to the low instructor-to-participant ratio. This ratio is among our limitations. While this low ratio makes it financially feasible for institutes to integrate the course into their curriculum and reduce the overall cost for participants, we remained concerned about the quality of delivering information and skills under such conditions. Our perspective shifted after surveying participants following their one-year internship. However, this survey is not an objective tool for assessment and can only assess the participant's points of view. Notably, our study should have assessed participants' performance immediately post-course, which we acknowledge as a limitation. To avoid this, we recommend adding an OSCE conducted separately from the course schedule on a separate day. This would robustly evaluate participants' skills shortly after the course completion, facilitating immediate and actionable feedback. Finally, our study did not track the subsequent career paths of the participants, missing an opportunity to evaluate the long-term influence of the course on career decisions and specialty selection. Looking to the future, it is imperative that research efforts focus on innovative ways to assess participant progress while simultaneously exploring various ways to reduce the financial burden on newly graduated medical students and institutes.

## Conclusions

In response to concerns about newly graduated physicians' competence during the transition to internship, we introduced the IPCC. This course addresses a critical gap in medical education. Our innovative timed-station methodology, inspired by the OSCE, actively engages participants in practical scenarios, boosting their skills and confidence. The one-year follow-up survey shows positive outcomes, with participants reporting practical skill application during internships. Future research should address limitations related to instructor-to-participant ratios and post-course OSCE assessment and explore cost-effective conducting methods.
